# Antioxidant, Anti-Inflammatory, and Analgesic Activities of* Agrimonia eupatoria* L. Infusion

**DOI:** 10.1155/2017/8309894

**Published:** 2017-04-12

**Authors:** Telmo N. Santos, Gustavo Costa, J. Pinto Ferreira, Joana Liberal, Vera Francisco, António Paranhos, Maria T. Cruz, M. Castelo-Branco, I. Vitória Figueiredo, M. Teresa Batista

**Affiliations:** ^1^Faculty of Pharmacy, University of Coimbra, Azinhaga de Santa Comba, 3000-548 Coimbra, Portugal; ^2^Center for Neurosciences and Cell Biology, University of Coimbra, Azinhaga de Santa Comba, 3000-548 Coimbra, Portugal; ^3^Escola Superior de Saúde Dr. Lopes Dias, Instituto Politécnico de Castelo Branco, Castelo Branco, Portugal; ^4^Institute for Biomedical Imaging and Life Sciences, University of Coimbra, Coimbra, Portugal

## Abstract

Agrimony (*Agrimonia eupatoria* L.) (Ae) is used in traditional medicine to treat inflammatory and oxidative related diseases. Therefore, this study focuses on the anti-inflammatory and analgesic potential of Ae infusion (AeI). Phenolic compounds characterization was achieved by HPLC-PDA-ESI/MS^*n*^. To evaluate antioxidant potential, 2,2-diphenyl-1-picrylhydrazyl (DPPH), superoxide anion, hydroxyl radical, and SNAP assays were used. In vitro anti-inflammatory activity of AeI was investigated in LPS-stimulated macrophages by measuring the NO production. In vivo anti-inflammatory activity was validated using the mouse carrageenan-induced paw edema model. Peripheral and central analgesic potential was evaluated using the acetic acid-induced writhing and hot-plate tests, respectively, as well as the formalin assay to assess both activities. The safety profile was disclosed in vitro and in vivo, using MTT and hematoxylin assays, respectively. Vitexin, quercetin* O*-galloyl-hexoside, and kaempferol* O*-acetyl-hexosyl-rhamnoside were referred to in this species for the first time. AeI and mainly AePF (Ae polyphenolic fraction) showed a significant antiradical activity against all tested radicals. Both AeI and AePF decreased NO levels in vitro, AePF being more active than AeI. In vivo anti-inflammatory and analgesic activities were verified for both samples at concentrations devoid of toxicity. Agrimony infusion and, mainly, AePF are potential sources of antiradical and anti-inflammatory polyphenols.

## 1. Introduction

*Agrimonia eupatoria* L., Rosaceae, commonly known as agrimony, is an erect, perennial herb, up to 100 cm high, mostly unbranched, with a cylindrical stem. The pinnate leaves are serrated and covered with soft hairs. Flowers are hermaphrodite with 5 yellow petals, arranged on slender, terminal spikes. The fruit is surrounded by several rows of soft, hook-shaped bristles [1].

The aerial parts of agrimony are used to prepare infusions, decoctions, or tinctures (hydroalcoholic extracts) in traditional medicine, due to their antioxidant, anti-inflammatory, astringent, and diuretic properties [2, 3]. Nonetheless, only a small number of scientific works have been carried out concerning these activities and the phytoconstituents involved. Aromatic acids, triterpenes, and tannins have been cited [4]. The presence of kaempferide, kaempferol, and their derivatives, such as kaempferol 3-*O*-glucoside, kaempferol 3-*O*-rhamnoside, and kaempferol 3-*O*-rutinoside, has also been reported [5, 6]. However, to our knowledge, little scientific information has been published about some of its phenolic compounds, namely, proanthocyanidins, and of the recently discovered compounds like glycosylated flavonoid phenolic acid esters, such as kaempferol 3-*O*-(2′′-*O*-acetyl-6′′-*p*-coumaroyl)-glucoside [3, 7]. Condensed tannins, generally designed by proanthocyanidins, are a group of phytochemicals that has been described as potent antioxidants. Usually, the scavenging activity of those flavan-3-ol polymers increases with the number of hydroxyls, especially if they are in* ortho* position on B-ring, and for their gallic acid esters. Antiradical activity also increases from monomers to trimers, though it decreases afterwards [3].

Flavonoids belong to an extensive group of polyphenols, and several biological activities, such as anti-inflammatory, antiulcer, anticancer, antiviral, antibacterial, antispasmodic, neuroprotective, antiatherosclerotic, and antithrombotic, have been attributed to these compounds. Oxidative stress and antioxidant defense have been associated with inflammatory, carcinogenic, and coronary diseases. The recognized antioxidant potential of flavonoids could therefore be responsible for their beneficial actions [8]. Moreover, anti-inflammatory properties, which are sometimes correlated with antioxidant activities, have also been verified for these compounds [9–11].

Extensive research within the last two decades has revealed that most chronic illnesses, including cancer, diabetes, and cardiovascular disease, are mediated through chronic inflammation, which currently constitutes one of the leading causes of mortality in western world [12]. Despite the fact that the mechanisms by which it happens have not been clarified yet, it is known that reactive species act as second messengers triggering the aberrant expression of inflammatory genes, such as cytokines, chemokines, cyclooxygenase-2 (COX-2), and inducible nitric oxide synthase (iNOS). It is also known that reactive species, ROS and RNS, can activate signal transduction cascades as well as inducing changes in transcription factors, which mediate immediate cellular inflammatory and stress responses [12]. The main pathway initiating this inflammatory process is the nuclear factor NF-kB signaling pathway [13]. Inflammation is usually associated with pain. In fact, many of the chemical mediators, namely, histamine, serotonin, bradykinin, prostaglandins, TNF-*α*, and protons, generated during inflammation, stimulate the nociceptive receptors responsible for peripheral pain [14]. Previous results demonstrated that an aqueous extract of* Agrimonia eupatoria* exhibited a prominent free radical-scavenging effect against DPPH radical [3, 15, 16] and also against ABTS radical cation [17]. In general, the increase of hydroxyl substitutions is correlated with a stronger antioxidant activity [18].

Folk medicine from different countries also reports the strong anti-inflammatory potential of* A. eupatoria* preparations [3], which was correlated with the reduction of TNF-*α*, IL-1*β*, and IL-6 production in mouse cell cultures [19]. Additionally, it was also previously reported that agrimony ameliorated the chronic ethanol-induced liver injury and that protection was likely due to the suppression of oxidative stress and* Toll-like *receptor- (TLR-) mediated inflammatory signaling [20].

In our ongoing work, we characterized* Agrimonia eupatoria* hydroalcoholic extracts and proved that they have pronounced antioxidant activity against reactive species, mainly due to specific flavonoids and/or condensed tannins, identified as flavonol, flavone, and flavan-3-ol derivatives [21, 22]. Accordingly, a recent study confirmed the presence of these compounds in agrimony and among them are proanthocyanidins, apigenin, luteolin, quercetin, and kaempferol derivatives, but also the catechin, chlorogenic acid, and* p*-coumaric acid were identified [23].

This study aims to characterize the antioxidant activity of the* Agrimonia eupatoria* L. infusion and a polyphenol-enriched fraction and to correlate this bioactivity with its phenolic profile. Besides, we aimed to assess the in vitro anti-inflammatory activity of both the infusion and the polyphenol-enriched fraction at concentrations devoid of toxicity. The anti-inflammatory and analgesic properties of these samples were validated using in vivo models, while the liver and kidney toxicities were monitored in order to support the traditional use of this medicinal plant.

## 2. Materials and Methods

### 2.1. Plant Material, Extract, and Fractioning

Aerial parts of* Agrimonia eupatoria* L. were provided by Segredo da Planta, Portugal (batch number 5870 and quality control data identifying no defects), identified by Dr. Célia Cabral and a voucher specimen (T. Batista 02009) was deposited at the Herbarium of Medicinal and Aromatic Plants, Faculty of Pharmacy, University of Coimbra. The extract (infusion) was prepared according to the usages in traditional medicine; in particular, 20 g of pulverized plant was infused in 600 mL of water, for 15 min. The infusion was then vacuum-filtered and washed with* n*-hexane (1 : 1) to eliminate the fat-soluble compounds. Next, a half of the infusion was concentrated in a Rotavapor at 30°C, frozen, and freeze-dried (AeI), while the other half was fractionated by repeated extraction with ethyl acetate (Merck, Darmstadt, Germany) (3 × 225 mL). The organic phases, containing polyphenols, were collected, dehydrated with anhydrous sodium sulfate, and concentrated with water, in a Rotavapor at 30°C, until all the ethyl acetate had been removed. Ethyl acetate fraction was frozen and freeze-dried (AePF).

### 2.2. HPLC-PDA-ESI/MS^*n*^

AeI and AePF were analyzed by a Surveyor liquid chromatograph equipped with a photodiodes (PDA) detector (Surveyor) and interfaced with a Finnigan LCQ (San Jose, CA, USA) mass spectrometer equipped with an API-ES ionization chamber. Separation was performed on an Spherisorb® ODS-2 C_18_ reverse phase column, 150 × 2.1 mm i.d. and size particle of 3 *μ*m, (Waters Corporation, Milford, Massachusetts, USA) and a Spherisorb ODS-2 guard cartridge C18 (10 × 4.6 mm i.d. and size particle of 5 *μ*m; Waters Corporation [Milford, Massachusetts, USA]) at 25°C, using 2% aqueous formic acid (A) and methanol (B) as mobile phase. The gradient profile used was 5–15% B (0–10 min), 15–30% B (10–15 min), 30–35% B (15–25 min), 35–50% B (25–35 min), 50–80% B (35–40 min), and 80% B (40–60 min), isocratically, at a flow rate of 0.2 mL/min. The first detection was done with the PDA detector in a wavelength range of 200–600 nm, followed by a second detection in the mass spectrometer.

Mass analyses were obtained in the negative ion mode. The mass spectrometer was programmed to perform three consecutive scans: full mass MS^1^ (*m/z* 160–1300), MS^2^ of the most abundant ion in MS^1^, and MS^3^ of the most abundant ion in MS^2^. Source voltage was 4.5 kV and capillary voltage and temperature were −10 V and 250°C, respectively. Nitrogen was used as sheath gas at flow rate of 20 arbitrary units. The normalized energy of collision was 45%, using helium as collision gas.

### 2.3. Antioxidant Activity

Free radical-scavenging activity was evaluated according to the method described by Blois, 1958 [24]. Aliquots of samples (100 *μ*L) were assessed by their reactivity with methanolic solution of 500 *μ*M DPPH (2,2-diphenyl-1-picrylhydrazyl) (500 *μ*L) (Sigma-Aldrich, Portugal) in the presence of 100 mM acetate buffer, pH 6.0 (1 mL). Reaction mixtures (3 mL) were kept for 30 min at room temperature and in the dark. The decreases in the absorbance were measured at 517 nm, in a Cintra 101, GBC® (Victoria, Australia) spectrophotometer. Butylhydroxytoluene (BHT) (Sigma-Aldrich, Portugal) was used as reference compound. Data treatment and statistical analysis were achieved using Microsoft® Excel 2013.

The assay was based on the superoxide-driven reduction of nitroblue tetrazolium (NBT) by photochemically reduced riboflavin [25]. Reaction mixtures (3 mL) contained phosphate buffer (16 mM, pH 7.8), 0.1 mM ethylenediaminetetraacetic acid (EDTA), 0.8 mM tetramethylethylenediamine (TEMED), 85 *μ*M nitroblue tetrazolium (NBT), 6 *μ*M riboflavin, and the appropriate volume of the samples. The assay was carried out at room temperature (22°C) under fluorescent lighting (20 W, 20 cm). Reaction was stopped by switching off the light and the addition of 50 *μ*L of 1 mg/mL superoxide dismutase (SOD). Absorbance was measured at 560 nm. Quercetin was used as reference compound.

The scavenging capacity for hydroxyl radical was evaluated using a deoxyribose degradation assay performed according to the procedure described by Payá et al. [26]. In a final volume of 1 mL, reaction mixtures contained 20 mM phosphate buffer (pH 7.4), 2.8 mM 2-deoxy-D-ribose, 104 *μ*M EDTA, 20 *μ*M FeCl_3_, 0.5 mM H_2_O_2_, 0.1 mM ascorbic acid, and the sample aliquots. Mixtures were kept at 37°C for 1 h and, then, the extent of deoxyribose degradation was measured by adding 1 mL of 1% thiobarbituric acid (TBA) (w/v) and 1 mL of 2.8% trichloroacetic acid (TCA) (w/v), followed by heating at 100°C for 20 min. After cooling, absorbance was measured at 532 nm. Quercetin was used as reference.

### 2.4. In Vitro Assays

Raw 264.7, a mouse leukaemic monocyte macrophage cell line from American Type Culture Collection, kindly supplied by Dr. Otília Vieira (Centro de Neurociências e Biologia Celular, Universidade de Coimbra, Coimbra, Portugal), was cultured in Iscove's Modified Dulbecco's Eagle Medium supplemented with 10% noninactivated fetal bovine serum, 100 U/mL penicillin, and 100 *μ*g/mL streptomycin at 37°C in a humidified atmosphere of 95% air and 5% CO_2_. Along the experiments, cells were monitored by microscope observation in order to detect any morphological change.

Assessment of metabolically active cells was performed using 3-(4,5-dimethylthiazol-2-yl)-2,5-diphenyl tetrazolium bromide (MTT) reduction colorimetric assay as previously reported [27]. Raw 264.7 cells (6 × 10^5^ cells/well) were plated (48-well plate) and allowed to stabilize for 12 h. Following this period, cells were either maintained in culture medium (control) or preincubated with the sample for 1 h and later activated with 1 *μ*g/mL LPS (Sigma Chemical Co., USA) for 24 h. After the treatments, a MTT solution (5 mg/mL in phosphate buffered saline) was added and cells incubated at 37°C for 15 min, in a humidified atmosphere of 95% air and 5% CO_2_. Supernatants were then removed and dark blue crystals of formazan solubilized with acidic isopropanol (0.04 N HCl in isopropanol). Quantification of formazan was performed using an ELISA automatic microplate reader (SLT, Austria) at 570 nm, with a reference wavelength of 620 nm. Data are expressed as mean ± SEM. Statistical significance was determined by one-way ANOVA, followed by Dunnett's post hoc test.

The production of nitric oxide (NO) was measured by the accumulation of nitrite in the culture supernatants, using a colorimetric reaction with the Griess reagent [28]. Briefly, 170 *μ*L of culture supernatants was diluted with equal volumes of the Griess reagent [0.1% (w/v) N-(1-naphthyl)-ethylenediamine dihydrochloride and 1% (w/v) sulphanilamide containing 5% (w/v) H_3_PO_4_] and maintained during 30 min, in the dark. The absorbance at 550 nm was measured in an automated plate reader (SLT, Austria). Culture medium was used as blank and nitrite concentration was determined from a regression analysis using serial dilutions of sodium nitrite as standard. Data are expressed as mean ± SEM. Statistical significance was determined by one-way ANOVA, followed by Dunnett's post hoc test.

The nitric oxide (NO) scavenging activity was evaluated by incubating the samples with 150 *μ*M of NO donor S-nitroso-N-acetylpenicillamine (SNAP; Tocris Bioscience, Bristol, UK), in Iscove's Modified Dulbecco's Eagle Medium (Sigma-Aldrich Química, Madrid, Spain). The concentration of the samples was chosen based on the EC_50_ concentration of DPPH radical-scavenging results. After 3 h, the nitrite levels in the medium were quantified by Griess method, as described above. Data are expressed as mean ± SEM. Statistical significance was determined by one-way ANOVA, followed by Dunnett's post hoc test.

### 2.5. In Vivo Assays

Male Wistar rats weighing 160–220 g and male mice weighing 25–30 g were obtained from Charles River (Barcelona, Spain). The animals were maintained with food and water ad libitum and kept at 22 ± 1°C with controlled 12-hour light/dark cycle at Faculty of Pharmacy, University of Coimbra. The animals were allowed to adapt to the laboratory for 7 days before testing. For evaluation of the anti-inflammatory activity, rats were fasted with free access to water at least 24 h prior to experiments. To study the analgesic activity of the samples, mice were fasted with free access to water at least 18 h prior to experiments. The research was conducted in accordance with the internationally accepted principles for laboratory animal use and care as found in Directive 2010/63/EU and the project was approved by the Portuguese Veterinary General Division.

*Agrimonia eupatoria* infusion (AeI) and its ethyl acetate fraction (AePF) were dissolved in distilled water and the administrated doses were calculated based on the traditionally used doses (extract of 5 g of plant in 150 mL of water), taking into account our yield of the extraction by infusion and by ethyl acetate fractioning process, as well as the conversion into animal doses, according to the FDA (Food and Drug Administration) guidelines, “Estimating the Safe Starting Dose in Clinical Trials for Therapeutics in Adult Healthy Volunteers” [29]. In order to examine a possible dose-dependent effect, two different doses were used: D1 and D2 (in the inflammation assay) and D2 and D3 (in both pain evaluation assays), according to the animal model used. During the experimental studies, procedures to reduce experimenter subjectivity and increase bias were followed.

#### 2.5.1. Anti-Inflammatory Activity

For the experimental evaluation of anti-inflammatory activity, the animals were anesthetized with a solution of sodium ketamine (Ketalar®, 50 mg/kg) purchased from Parke-Davis, Pfizer (Seixal, Portugal) and chlorpromazine (Largactil®, 2.3 mg/kg) from Rhône-Poulenc Rorer, Laboratório Vitória SA (Amadora, Portugal).

The carrageenan-induced rat paw edema test [27] was used to evaluate the in vivo acute anti-inflammatory activity of the AeI and AePF, employing diclofenac sodium as reference drug. Rat paw edema was assessed by the volume displacement method resorting to a digital Plethysmometer (model LE7500, Panlab, Barcelona, Spain). Before the attainment of the experiment, a preassay was carried out in order to ascertain in which time occurs the maximum of edema volume, in other words, the maximum of inflammation. For that, a subplantar injection of 0.1 mL of 1% solution of carrageenan in saline, administered to the right hind footpad of each animal, was carried out, and the paw volume was measured in intervals of 30 min. in a period of 7 h. The results of this experiment showed us that the maximum value of paw edema occurs 4 h after the carrageenan administration, being this the period of time used for the following experiment.

The rats were divided into four groups (*n* = 6–8): carrageenan negative control group received water 0.5 *μ*L/g p.o., positive control group received diclofenac sodium 10 mg/kg i.p., and test groups received orally AeI (99.59 mg/kg, D1, and 199.18 mg/kg, D2) and AePF (18.12 mg/kg, D1, and 36.24 mg/kg, D2) reconstituted in water. Negative control and test groups, respectively, were dosed with the vehicle and samples, 1 h, and positive control group was dosed with diclofenac sodium, 30 min before the administration of a subplantar injection of 0.1 mL of 1% solution of carrageenan in saline, to the right hind footpad of each animal. The potential anti-inflammatory was calculated 4 h after carrageenan administration and was expressed as percentage of edema inhibition, for the treated animals, in relation to the carrageenan control group. The percentage of edema inhibition was calculated according to the following equation: % edema inhibition = 1 − (*V*_*t*_/*V*_*c*_) × 100, where *V*_*t*_ is the mean variation in paw volume in rats treated with the AeI, AePF, or diclofenac sodium and *V*_*c*_ is the mean variation in paw volume in the carrageenan control group. Data are expressed as mean ± SEM. Statistical significance was determined by one-way ANOVA, followed by Tukey's post hoc test.

#### 2.5.2. Central Analgesic Activity

The hot-plate test was used to evaluate the central analgesic activity of the AeI and AePF by measuring the reaction time according to the method described by Eddy and Leimbach [30]. Morphine was used as reference drug. The mice were placed individually on a hot-plate set at 55 ± 1°C (model LE7406, PanLab, Barcelona, Spain). Reaction time, measured in seconds, was recorded when the animals licked their forepaws and jumped. The baseline was considered as being the reaction time before treatment and was measured 24 h previous to the experiment. Mice were then divided randomly into five groups (*n* = 6–8): positive control group received morphine hydrochloride 10 mg/kg i.p. and test groups received orally the AeI (199.18 mg/kg, D2, and 398.26 mg/kg, D3) and the AePF (36.24 mg/kg, D2, and 72.48 mg/kg, D3) reconstituted in water. Test groups were dosed 1 h and positive control group was dosed 30 min before placement on the hot plate for recording of reaction time to temperature. The cut-off time was set at 30 s to protect the animals. The percentage of analgesic capacity was calculated by using the formula: % analgesic capacity = 1 − (*T*_*b*_/*T*_*t*_) × 100, where *T*_*b*_ is the mean reaction time recorded as baseline and *T*_*t*_ is the mean reaction time recorded after treatment with morphine or with the sample. Data are expressed as mean ± SEM. Statistical significance was determined by one-way ANOVA, followed by Tukey's post hoc test.

#### 2.5.3. Peripheral Analgesic Activity

The acetic acid-induced writhing test was carried out according to the method previously described by Collier et al. [31]. The test was used to evaluate the peripheral analgesic activity of the AeI and AePF, employing diclofenac sodium as reference drug. Acetic acid at 0.6% in saline was administered i.p. 0.1 mL/10 g b.w. The number of writhes, a response consisting of the contraction of the abdominal wall followed by hind limb extension, was counted during continuous observation of the animals for 30 min, starting the count 5 min after the injection. The animals were divided into six groups (*n* = 6–8): negative control group received water 0.5 *μ*L/g p.o., positive control group received diclofenac sodium 10 mg/kg i.p., and test groups received the AeI and AePF at the doses referred to above. Negative control and test groups were dosed 1 h and positive control group was dosed 30 min before the acetic acid injection. A significant reduction in the number of writhes by any drug was considered as a positive analgesic response. Percent analgesic capacity was calculated by using the formula: % analgesic capacity = 1 − (*W*_*t*_/*W*_*c*_) × 100, where *W*_*t*_ is the average number of writhes in test and positive control groups and *W*_*c*_ is the average number of writhes in the negative control group. Data are expressed as mean ± SEM. Statistical significance was determined by one-way ANOVA, followed by Tukey's post hoc test.

#### 2.5.4. Analgesic Activity

The formalin test was carried out according to the method previously described by Hunskaar and Hole [32]. The test was used to evaluate either the peripheral or the central analgesic activity of the sample, employing, respectively, diclofenac sodium or morphine hydrochloride as reference drugs. Mice were divided into four groups (*n* = 6–8): a test group (72.48 mg/kg of the AePF p.o.), a negative control group (water p.o.), an early phase positive control group (morphine hydrochloride 10 mg/kg i.p.), and a late phase positive control group (diclofenac sodium 10 mg/kg i.p.). After 1 hour, for the test group and the negative control group, or 30 min, for the positive controls, the animals were subcutaneously injected with 20 *μ*L of 1% formalin in 0.9% saline into the dorsal hind paw and placed immediately inside a transparent box for observation. The duration of paw licking was determined within 0–5 min (early phase) and 20–40 min (late phase) after the formalin injection. The time spent licking, in seconds, was noted.

The analgesic capacity (in percentage) was calculated by using the formula: % analgesic capacity = 1 − (*W*_*t*_/*W*_*c*_) × 100, where *W*_*t*_ is the average time spent licking in test and positive control groups and *W*_*c*_ is the average time spent licking in the negative control group. Data are expressed as mean ± SEM. Statistical significance was determined by one-way ANOVA, followed by Tukey's post hoc test.

Five hours after administration of the samples, the rats used in the paw edema test were sacrificed. Livers and kidneys were collected, cut into transverse fragments (0.5 cm), and immersed in a 10% solution of formaldehyde in a 24 h period. The pieces were processed into paraffin and stored at −20°C. The fragments were cut in 4 *μ*m pieces in a Shandon (AS 325) microtome and stained with hematoxylin (cell nuclei, blue) and eosin (cytoplasm, pink/red). The resulting images were obtained and observed (20x magnification in the original) in a Nikon (Eclipse E600) microscope.

## 3. Results and Discussion

In the present study, the main polyphenols of* Agrimonia eupatoria* aerial part infusion were identified, the results demonstrating their contribution for the antioxidant, anti-inflammatory, and analgesic activities.

### 3.1. HPLC-PDA-ESI/MS^*n*^

**Figure 1:**
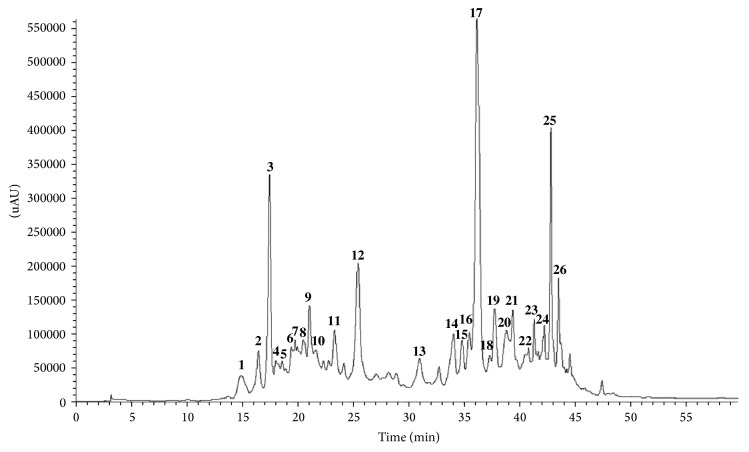
HPLC profile of* Agrimonia eupatoria* polyphenol-enriched ethyl acetate fraction (AePF). The chromatographic profile was registered at 280 nm. For experimental conditions, see Materials and Methods.

HPLC profiles were recorded at 280 nm for all samples, Figure 1 showing the HPLC profile from* Agrimonia eupatoria* polyphenol-enriched ethyl acetate fraction (AePF). Absorption maxima and ultraviolet profiles obtained with the diode array detector allowed most peaks to be identified as flavan-3-ols (**1**–**10**), quercetin (**14**,** 17**-**18**), kaempferol (**21**,** 23**–**26**), apigenin (**13**,** 15**,** 19,** and** 22**), and luteolin (**16**) derivatives and, in lesser extent, agrimoniin (**11**),* p*-coumaric acid (**12**), and ellagic acid (**20**).  Table 1 summarizes retention times, UV spectra maxima, MS^*n*^ fragments of relevant peaks and, on this basis, the tentative identification of the polyphenols present in the samples.

HPLC-PDA-ESI/MS^*n*^ analysis of AePF revealed the presence of procyanidins and flavonoids as the most relevant compounds of this fraction. Phenolic acids have been identified, namely,* p*-coumaric acid and ellagic acid, as described previously [21]. Many flavonoids were characterized by both PDA and MS detectors. Isovitexin, hyperoside [19], isoquercitrin, tiliroside [6], and other flavones and flavonols derivatives were found in this plant. To our knowledge, vitexin, quercetin* O*-galloyl-hexoside, and kaempferol* O*-acetyl-hexosyl-rhamnoside were found in this species for the first time. Interestingly, antiradical and/or anti-inflammatory activities have been reported for these three flavonoids [33–35].

### 3.2. Antioxidant Activity

As shown in Table 2,* Agrimonia eupatoria* infusion (AeI) and its ethyl acetate fraction (AePF) exerted a significant scavenging effect, reacting with the DPPH free radical. AePF was the most potent sample with an EC_50_ of 4.60 *μ*g/mL, followed by AeI with an EC_50_ value of 12.80 *μ*g/mL.

Superoxide anion scavenging activity was observed for two samples, the most active being the AePF, which evidenced an EC_50_ of 3.34 *μ*g/mL. AeI exhibited a higher EC_50_, 13.59 *μ*g/mL (Table 2).

AePF was the most potent sample concerning the hydroxyl radical-scavenging effect, showing an EC_50_ of 90.97 *μ*g/mL. AeI showed an EC_50_ value of 126.99 *μ*g/mL (Table 2).

Concerning the antioxidant bioactivity, our results demonstrated that AePF was more active against DPPH, superoxide, and hydroxyl radical than the infusion. Therefore, the ethyl acetate extraction proved to be an efficient technique to extract compounds that confer antioxidant properties to* Agrimonia eupatoria* infusion. In fact, the effects of AePF on DPPH and superoxide anion were, respectively, similar to that of the BHT, a synthetic antioxidant, and higher than quercetin, a potent natural antioxidant. These results are in accordance with previously described data for kaempferol, quercetin, and tiliroside, exhibiting important DPPH and superoxide scavenging activities [35–37]. On the other hand, weaker activities against the hydroxyl radical were observed, corroborating what is cited in literature.

### 3.3. In Vitro Assays

**Figure 2:**
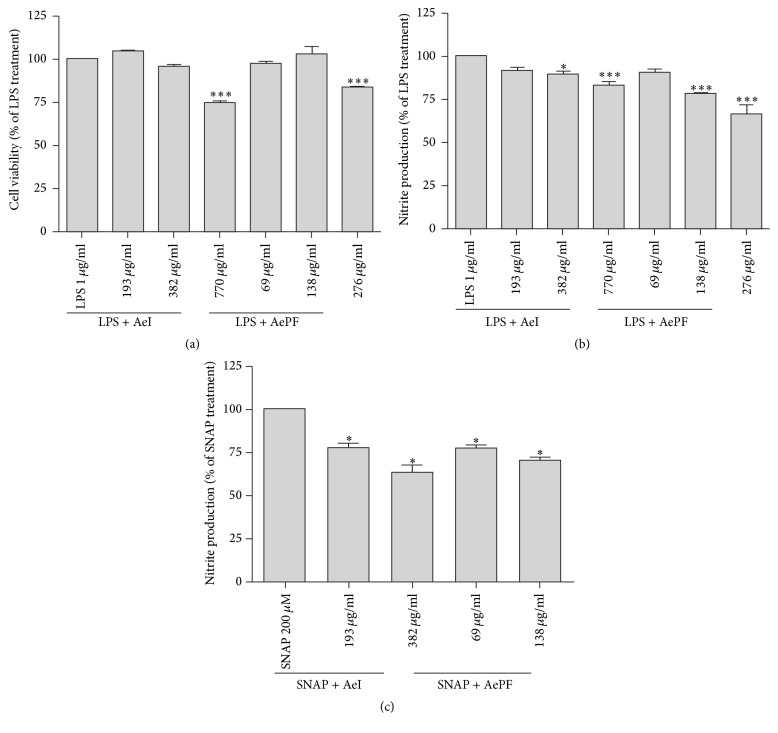
(a) Cell viability (% of LPS) of Raw 264.7 cells incubated with AeI (193, 382 and 770 *μ*g/mL) or AePF (69, 138 and 276 *μ*g/mL) for 1 h, followed by incubation with LPS for 24 h. Data represent mean ± SEM of 3 independent assays. ^*∗∗∗*^*P* < 0.001, compared to control with LPS. (b) Nitrite production (% of LPS) of Raw 264.7 cells after incubation with AeI (193, 382, and 770 *μ*g/mL) or AePF (69, 138, and 276 *μ*g/mL) for 1 h, followed by incubation with LPS for 24 h. Data represent mean ± SEM of 3 independent assays. ^*∗∗∗*^*P* < 0.001 and ^*∗*^*P* < 0.05, compared to control with LPS. (c) Nitrite production (% of SNAP) after incubation of SNAP with AeI (193 and 382 *μ*g/mL) or AePF (69 and 138 *μ*g/mL) for 3 h. Data represent mean ± SEM of 3 independent assays. ^*∗*^*P* < 0.05, compared to control with SNAP.

The cytotoxic effect of AeI and AePF on RAW 264.7 macrophages was reported to percentage of lipopolysaccharide (LPS) showing that only the higher concentrations tested for AeI (770 *μ*g/mL) and AePF (276 *μ*g/mL) decrease the cell viability (Figure 2(a)).

The effect on NO production was analyzed by measuring nitrite accumulation in the culture medium of macrophages stimulated with LPS. As shown in Figure 2(b), incubation of macrophages with LPS, for 24 h, resulted in a significant increase in nitrite production compared to the control. Taking into account the toxicity of AeI and AePF for the concentrations 770 *μ*g/mL and 276 *μ*g/mL, respectively, which was discussed in the previous section, inhibition of NO production was only considered for nontoxic concentrations of the samples. Therefore, the AeI and AePF, for concentrations of 382 *μ*g/mL and 138 *μ*g/mL, reduced NO production relatively to LPS without detrimental effects to cells (11.34% and 22.46% of inhibition, resp.).

Only the samples which did not affect cell viability on the MTT assay were tested in the SNAP method used to evaluate the NO-scavenging capacity of the samples. For the concentrations tested, both AeI and AePF decreased the levels of nitrites in the culture medium (Figure 2(c)). AeI showed to significantly inhibit the SNAP activity by 36.67%, at a concentration of 382 *μ*g/mL. AePF decreased nitrites levels in a greater extent, with an inhibition of 29.46%, at a concentration of 138 *μ*g/mL.

Nitric oxide is a proinflammatory mediator involved in inflammatory-related diseases [38]. Therefore, a decrease in nitric oxide levels is an important strategy to ameliorate inflammation. Both AeI and AePF slightly decreased nitric oxide levels in vitro. In the SNAP assay, both samples (AeI and AePF) showed NO-scavenging properties with no statistically significant differences between their equivalent doses. This suggests that the mechanism behind nitrite levels reduction observed on the macrophages culture may be due to the NO-scavenging effect of the compounds present in AeI and AePF.

### 3.4. In Vivo Assays

#### 3.4.1. Anti-Inflammatory Activity

**Figure 3:**
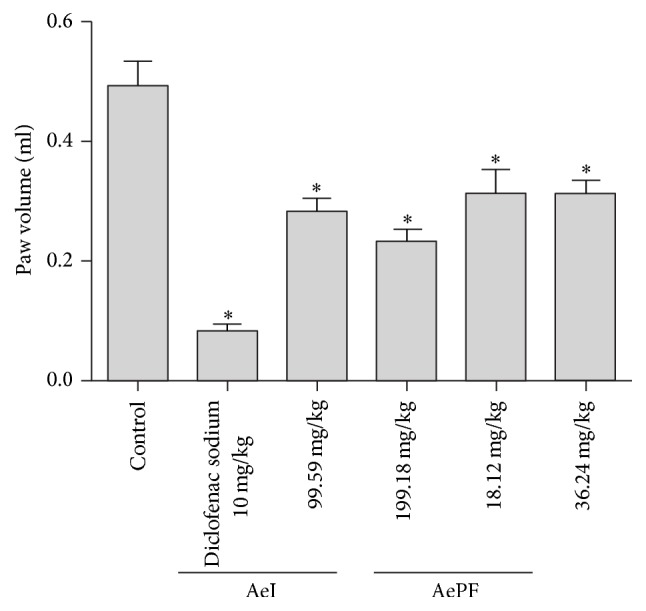
Carrageenan-induced rat paw edema test. Results were obtained by oral administration of an aqueous extract at doses 99.59 mg/kg b.w (AeID1) and 199.18 mg/kg b.w. (AeID2) and ethyl acetate fraction of the aqueous extract at doses 18.12 mg/kg b.w. (AePFD1) and 36.24 mg/kg b.w. (AePFD2) and 10 mg/kg (i.p.) of diclofenac sodium. Each value is mean ± SEM. of 6–8 rats. Statistical differences between the treated and the control groups were determined by ANOVA followed by Tukey's test. ^*∗*^*P* < 0.05 compared with control.

Carrageenan-induced paw edema was significantly reduced 4 h after the administration of the samples. The infusion reduced the edema by 43.2% (AeID1) and 52.2% (AeID2) and the polyphenol-enriched fraction reduced by 34.6% (AePFD1) and 35.4% (AePFD2) (Figure 3).

#### 3.4.2. Central Analgesic Activity

**Figure 4:**
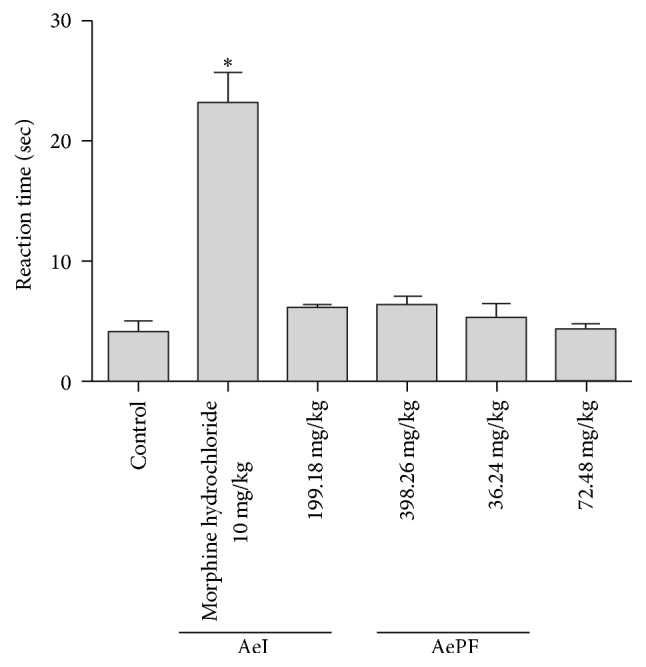
Hot-plate test. Results were obtained by oral administration of aqueous extract at doses 199.18 mg/Kg b.w (AeID2) and 398.26 mg/Kg b.w. (AeID3) and ethyl acetate fraction of the aqueous extract at doses 36.24 mg/Kg b.w. (AePFD2) and 72.48 mg/Kg b.w. (AePFD3) and 10 mg/Kg (i.p.) of morphine hydrochloride. Each value is the mean ± SEM of 6–8 rats. Statistical differences between the treated and the control groups were determined by ANOVA followed by Tukey's test. ^*∗*^*P* < 0.05 compared with control.

The results reveal that the mice treated with both doses of agrimony did not show any significant increase in the reaction time when in contact with the hot plate (Figure 4). In fact, at AeID2, the mean reaction time (±SEM) was 5.84 (±0.50) and at AeID3 was 6.20 (±0.77). For AePFD2, the mean reaction time (±SEM) was 5.17 (±1.18) and at AePFD3 was 4.16 (±0.49). The mice treated with morphine showed a marked increase in the reaction time to 23.05 (±2.72).

#### 3.4.3. Peripheral Analgesic Activity

**Figure 5:**
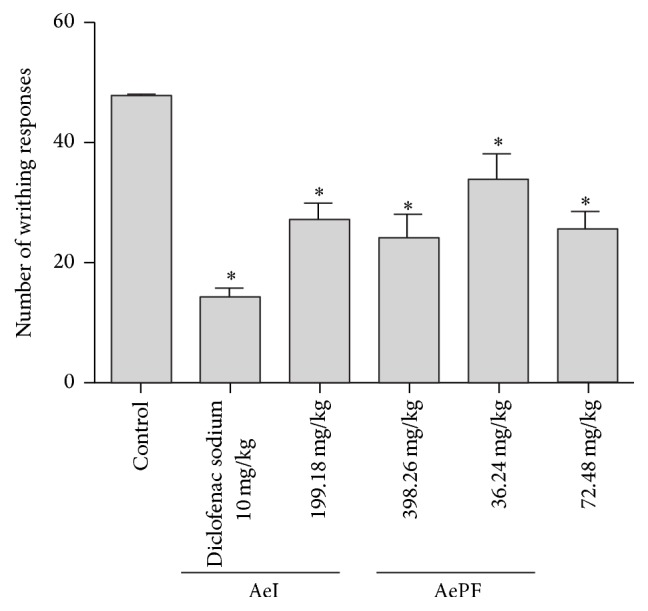
Acetic acid-induced writhing test. Results were obtained by oral administration of aqueous extract at doses 199.18 mg/Kg b.w (AeID2) and 398.26 mg/Kg b.w. (AeID3) and ethyl acetate fraction of the aqueous extract at doses 36.24 mg/Kg b.w. (AePFD2) and 72.48 mg/Kg b.w. (AePFD3) and 10 mg/Kg (i.p.) of diclofenac sodium. Each value is the mean ± SEM of 6–8 rats. Statistical differences between the treated and the control groups were determined by ANOVA followed by Tukey's test. ^*∗*^*P* < 0.05 compared with control.

The results presented in Figure 5 show that agrimony produces an inhibition of abdominal writhing induced by acetic acid in mice. The reference drug induced an inhibition of 71.3%. The mice treated with AeID2 and AeID3 showed a reduction of 43.5% and 49.8% on the average number of abdominal constrictions, respectively, whereas, with AePFD2 and AePFD3, the reduction was, respectively, 29.2% and 46.8%.

#### 3.4.4. Analgesic Activity

In the formalin test, only the AePFD3 dose was tested. It did not show any relevant effect in the early phase of pain response but induced a reduction of 32.5% in time spent licking on late phase (Table 3).

**Figure 6:**
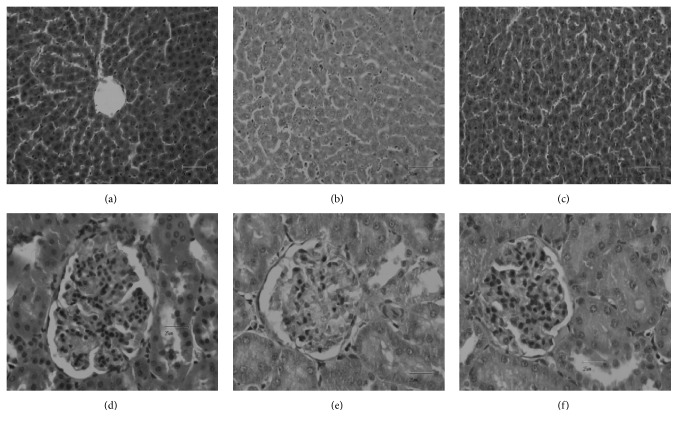
Histological analysis of liver and kidney. Staining with hematoxylin; 20x magnification in the original. (a–c) Liver tissues obtained from control animal (a); animal treated with reference drug (b); animal treated with AePF (c). (d–f) Renal tissues obtained from control animal (d); animal treated with reference drug (e); animal treated with AePF (f).

The liver and renal tissues of animals treated with the samples did not show differences resulting from toxic effects, comparatively to the animals of the control group (Figure 6).

In the experimental models used in in vivo assays, the samples presented a significant effect for the carrageenan-induced rat paw edema reduction. Also in this case, there are no significant differences between the equivalent doses of AeI and AePF. This is in accordance with the above results for the SNAP assay. Besides, although there is a tendency for a reduction on the inflammation values with the increased dose of AeI, there are no statistically significant differences between AeID1 and AeID2 and between AePFD1 and AePFD2. This may suggest that, for the tested concentrations, the anti-inflammatory activity of the compounds has already reached their maximum plateau. This hypothesis is supported by the fact that the doses used were calculated based on those among the highest one referred to in traditional medicine.

Diclofenac sodium is a common nonsteroid anti-inflammatory drug (NSAID) well known for its anti-inflammatory and analgesic activities. As agrimony presented ability to inhibit the swelling of the animal's paw, this suggests that its anti-inflammatory effect may be related to the inhibition of the synthesis and release of various inflammatory mediators and to its antioxidant activity and reactive oxygen and nitrogen species scavenging properties, eventually, acting as an inhibitor of polymorphonuclear leukocytes' action, thus inhibiting the release of reactive oxygen and nitrogen [39, 40].

A significant superoxide anion and NO-scavenging capacity was detected for AeI and for AePF. This antioxidant activity could be responsible for the anti-inflammatory properties of* Agrimonia eupatoria*. Superoxide anion is an inflammatory mediator and it reacts with NO originating peroxynitrite, which is a powerful oxidant that is also involved in the inflammatory processes. This inflammatory mediator acts through different pathways: inhibition of cytokine release and peroxynitrite formation and inhibition of neutrophils infiltration [41, 42]. Therefore, the significant superoxide anion scavenging activity observed may also contribute to the anti-inflammatory activity observed in the in vitro and in vivo assays.

The acetic acid-induced writhing model induces the release of endogenous mediators such as histamine, serotonin, bradykinin, acetylcholine, substance P, and prostaglandins that are involved in both proinflammatory and peripheral pain mechanisms. This model of pain is, therefore, sensitive to the action of NSAIDs [43, 44]. For* Agrimonia eupatoria*, the results suggest analgesic properties through peripheral mechanisms. This fact is probably due to the anti-inflammatory potential of agrimony. As these two physiologic processes share many molecular mediators and mechanisms [14], in some cases, their inhibition is concomitant. Actually, this type of anti-inflammatory mechanism has already been described in another species within the same genus,* Agrimonia pilosa* [45, 46].

Regarding central analgesia, infusion and fraction of agrimony showed no antinociceptive activity, not increasing the animals' reaction time on the hot plate. This supports the hypothesis above for the shared mechanism between analgesic and anti-inflammatory effects.

In the formalin test, the injection of formalin induces a biphasic pain reaction in mice, the early and the late phases [47]. The results are quantified as the time that the animals spend licking the paw [48]. The absence of antinociceptive effect of the AeI and AePF on early phase corroborates the above data, suggesting that its analgesic properties are not related to a modulation on the central nervous system. On the other hand, the reduction of the time spent licking on late phase supports the analgesia through peripheral mechanisms.

The ethyl acetate fractioning process allowed us to obtain a fraction with higher concentration of polyphenols. The fact that there are no significant differences between the anti-inflammatory and analgesic effects of AeI and AePF when comparing their equivalent doses, that is, doses with the same concentration of the compounds that are common to AeI and AePF, suggests that the main compounds responsible for these activities are probably the polyphenols of this fraction.

There are no signs of toxicity in the liver or kidneys tissues, when comparing the histopathological images of the tissues obtained from the control animals with the ones treated with the infusion and its fraction. Although further studies about this subject are needed, this data suggest an absence of systemic toxic effects of the samples at concentrations with therapeutic potential.

## 4. Conclusions

Antioxidant, anti-inflammatory, and peripheral analgesic activities were observed for both the* Agrimonia eupatoria* infusion and/or its polyphenol-enriched fraction at nontoxic doses for the liver and the kidneys. The polyphenol-enriched fraction, essentially constituted by procyanidins and flavonoids, namely, quercetin, kaempferol, apigenin, and luteolin derivatives, was very active for all assays performed, leading to the conclusion that polyphenols have a very important role in these properties of* Agrimonia eupatoria*.

The results of the present work corroborate the traditional use of the* Agrimonia eupatoria* infusion as antioxidant and anti-inflammatory and suggests that its polyphenols contribute to this activity, which may be helpful in developing novel and beneficial antioxidants, anti-inflammatory, and/or peripheral analgesic agents. In addition, isoquercetin, tiliroside, and kaempferol* O*-acetyl-hexosyl-*O*-rhamnoside should be considered as lead molecules for designing new pharmacophores, to be applied to the treatment of oxidation- and inflammation-related pathologies.

## Figures and Tables

**Table 1:** Phenolic characterization of AePF from *Agrimonia eupatoria*, by HPLC-PDA-ESI/MS^*n*^.

Peak	Rt (min)	Tentative identification	*λ*max (nm)	|M − H|^−^ (*m/z*)	MS^2^	MS^3^
					[*m/z* (relative abundance, %)]	
**1**	14.94	Procyanidin dimer	279	577	451 (18), **425 (100)**, 407 (84)	**407 (100)**, 273 (5)
**2**	16.48	Procyanidin trimer	278	865	739 (36), 713 (34), **695 (100)**, 577 (49), 543 (15), 425 (20), 407 (21), 405 (14)	677 (39), 543 (69), **525 (100)**
**3**	17.48	Catechin^**∗**^	279	289	**245 (100)**, 205 (41), 203 (16), 179 (7)	227 (34), 213 (11), **203 (100)**, 175 (17), 174 (11)
**4**	18.07	Procyanidin dimer	279	577	451 (22), **425 (100)**, 407 (83), 289 (26)	**407 (100)**, 381 (4), 245 (5)
**5**	18.25	Procyanidin trimer	279	865	739 (20), **695 (100)**, 587 (36), 577 (46), 575 (23), 451 (23), 425 (14), 407 (20)	**525 (100)**, 203 (53)
**6**	18.94	Procyanidin tetramer	279	1153	1057 (51), 983 (51), 965 (68), 863 (39), 813 (84), 713 (41), **695 (100)**, 617 (92)	—
**7**	19.78	Procyanidin tetramer	279	1153	917 (99), 863 (88), 813 (86), **489 (100)**	—
**8**	20.01	Procyanidin trimer	280	865	739 (13), 738 (14), **695 (100)**, 587 (23), 577 (38), 569 (10), 543 (33), 407 (29)	677 (30), 585 (50), 525 (88), **407 (100)**
**9**	21.10	Procyanidin dimer	279	577	451 (12), **425 (100)**, 407 (75)	—
**10**	21.65	Procyanidin trimer	280	865	739 (34), 738 (11), **695 (100)**, 587 (28), 577 (51), 569 (20), 561 (24), 543 (21), 451 (17), 407 (22)	525 (83), **407 (100)**
**11**	23.01	Agrimoniin	258sh, 282	935^§^	899 (3), **633 (100)**, 523 (2), 463 (5), 301 (93)	**301 (100)**
**12**	25.47	*p*-Coumaric acid^**∗**^	298sh, 310	—	—	—
**13**	31.01	Apigenin 8-C-glucoside (vitexin)	270, 331	431	341 (3),** 311 (100)**	293 (3), **283 (100)**, 239 (4)
**14**	34.07	Quercetin *O*-galloyl-hexoside	259sh, 265, 342	615	**463 (100)**, 301 (9), 300 (3)	**301 (100)**, 300 (93), 296 (2), 283 (2), 179 (1)
**15**	34.85	Apigenin 6-C-glucoside (isovitexin)^**∗**^	270, 333	431	413 (17), 395 (1), 383 (2), 355 (1), 341 (54), **311 (100)**	**283 (100)**
**16**	35.50	Luteolin 7-*O*-glucoside (Cynaroside)^**∗**^	268, 295sh, 348	447	327 (6), **285 (100)**	**285 (100)**, 257 (85), 242 (37), 241 (42), 217 (60), 198 (62)
**17**	36.19	Quercetin 3-O-glucoside (isoquercetin)^**∗**^	256, 266sh, 355	463	**301 (100)**, 179 (3)	273 (12), **271 (100)**, 257 (15), 255 (61), 179 (98)
**18**	37.35	Quercetin *O*-malonyl-hexoside	259, 267sh, 348	549	**505 (100)**, 464 (1), 387 (1)	463 (27), 342 (10), **301 (100)**
**19**	38.27	Apigenin 7-O-glucoside (apigetrin)^**∗**^	258sh, 265, 331	431	311 (11), **269 (100)**, 268 (22)	**159 (100)**
**20**	38.77	Ellagic acid	257, 351	301	**257 (100)**, 179 (2), 151 (2)	**179 (100)**, 151 (60)
**21**	39.42	Kaempferol 3-O-glucoside (astragalin)^**∗**^	265, 346	447	327 (12), **285 (100)**, 255 (15)	257 (16), 256 (21), **255 (100)**, 227 (13)
**22**	40.86	Apigenin *O*-glucuronide	258sh, 264, 331	445	307 (10), **269 (100)**, 175 (20)	225 (40), 185 (63)
**23**	41.53	Kaempferol *O*-malonyl-hexoside	257sh, 264, 340	533	**489 (100)**	**285 (100)**
**24**	42.31	Kaempferide *O*-rhamnoside	265, 311, 348	445	**298 (100)**, 283 (5)	**269 (100)**, 207 (22), 191 (7), 187 (4), 163 (24), 148 (6)
**25**	42.92	Kaempferol O-*p*-coumaroyl-glucoside (tiliroside)	267, 290sh, 315, 349	593	447 (15), 429 (2), 323 (1), **285 (100)**, 284 (3), 257 (3)	267 (32), 257 (82), **243 (100)**, 211 (57)
**26**	43.56	Kaempferol *O*-acetyl-hexosyl-*O*-rhamnoside	267, 294sh, 315, 348	635	575 (21), 489 (20), 431 (11), 349 (5), **285 (100)**, 255 (3)	**257 (100)**, 255 (42), 240 (8), 213 (9), 183 (9), 151 (21)

sh: shoulder; ^§^|M − 2H|^2−^; ^**∗**^confirmed by commercial reference compounds.

**Table 2:** Free radical scavenging effect of AeI and AePF from *Agrimonia eupatoria*.

Sample	EC_50_^a^		
	DPPH radical	Superoxide anion	Hydroxyl radical
AeI	12.80 ± 0.05	13.59 ± 1.03	126.99 ± 11.97
AePF	4.60 ± 0.05	3.34 ± 0.20	90.97 ± 8.29
BHT	4.67 ± 0.05	—	—
Quercetin	—	5.34 ± 0.43	15.74 ± 1.31

^a^Amount of the samples (*µ*g/mL of reaction mixture) that decreased 50% of the absorbance values as compared to the negative control. Each value is the mean ± SD of the three replicates.

**Table 3:** Effect of AePFD3 on the time that mice spent licking their hind paw during early and late phase of formalin test.

Group	Dose (mg/kg)	Time spent licking (0–5 min)	Time spent licking (20–40 min)	Pain reduction (%)
Control	—	56.00 ± 4.05	183.80 ± 16.07	—
Reference	10	3.67 ± 0.76^*∗*^	30.40 ± 5.77^*∗*^	98.4
AePFD3	72.48	48.17 ± 3.19	124.00 ± 6.35^*∗*^	32.5

Reference: morphine hydrochloride (10 mg/kg b.w.) for early phase or diclofenac sodium (10 mg/kg b.w.) for late phase; AePFD3: ethyl acetate fraction of the aqueous extract at dose 72.48 mg/kg b.w. Each value is the mean ± SEM of 6–8 rats. Statistical differences between the treated and the control groups were determined by ANOVA followed by Tukey's test. ^*∗*^*P* < 0.05 compared with control.
